# Glycemic control and survival in peritoneal dialysis patients with diabetes: A 2-year nationwide cohort study

**DOI:** 10.1038/s41598-019-39933-5

**Published:** 2019-03-01

**Authors:** Masanori Abe, Takayuki Hamano, Junichi Hoshino, Atsushi Wada, Shigeru Nakai, Ikuto Masakane

**Affiliations:** 1 The Committee of Renal Data Registry, Japanese Society for Dialysis Therapy, Tokyo, Japan; 2 Division of Nephrology, Hypertension and Endocrinology, Department of Internal Medicine, Nihon University School of Medicine, Tokyo, Japan; 3 Department of Inter-Organ Communication Research in Kidney Disease, Osaka University Graduate School of Medicine, Osaka, Japan; 4 Nephrology Center, Toranomon Hospital, Tokyo, Japan; 5 Department of Nephrology, Kitasaito Hospital, Asahikawa, Japan; 6 Department of Clinical Engineering, Fujita Health University, Aichi, Japan; 7 Yabuki Hospital, Yamagata, Japan

## Abstract

For glycemic control in patients with diabetes on peritoneal dialysis (PD), the level of glycated albumin (GA) associated with mortality is unclear. Accordingly, we examined the difference in the association of GA and glycated hemoglobin (HbA1c) with 2-year mortality in a Japanese Society for Dialysis Therapy cohort. We examined 1601 patients with prevalent diabetes who were on PD. Of these, 1282 had HbA1c (HbA1c cohort) and 725 had GA (GA cohort) measured. We followed them for 2 years from 2013 to 2015 and used Cox regression to calculate adjusted hazard ratios (HRs) and 95% confidence intervals (CIs) for 2-year mortality after adjusting for potential confounders in each cohort. No significant association was found between HbA1c levels and all-cause death HRs before and after adjustment for confounders in the HbA1c cohort. In contrast, the adjusted all-cause death HRs and 95% CIs for GAs < 12.0%, 12.0–13.9%, 16.0–17.9%, 18.0–19.9%, 20.0–21.9%, and ≥22.0%, compared with 14.0–15.9% (reference), were 1.56 (0.32–7.45), 1.24 (0.32–4.83), 1.32 (0.36–4.77), 2.02 (0.54–7.53), 4.36 (1.10–17.0), and 4.10 (1.20–14.0), respectively. In the GA cohort, GA ≥ 20.0% was significantly associated with a higher death HR compared with the reference GA. Thus, GA ≥ 20.0% appears to be associated with a decrease in survival in diabetic patients on PD. There were no associations between HbA1c levels and 2-year mortality in PD patients.

## Introduction

Erythrocytes have a shorter life span than normal in dialysis patients, and blood loss and bleeding may also occur during hemodialysis (HD). This can lead to anemia requiring treatment with erythropoiesis-stimulating agents (ESAs), which falsely lowers the glycated hemoglobin (HbA1c) level by raising the proportion of young erythrocytes. As a result, HbA1c levels tend to be low in dialysis patients and glycemic control may be overestimated. In contrast, glycated albumin (GA) level is not significantly associated with erythrocyte life span, hemoglobin level, or ESA dose in patients with diabetes undergoing HD^1–4^. Thus, GA could be a more robust indicator of glycemic control than HbA1c in patients with diabetes on HD. Even so, the 2012 Kidney Disease Outcomes Quality Initiative (KDOQI) Clinical Practice Guideline for Diabetes does not recommend GA as a first-line tool for assessing glycemic control^5^. In patients with no history of cardiovascular events, survival was reported to be significantly longer in those with GA < 20.0% than that in those with GA 20.0–24.5 or >24.5%^6^. Based on these results, the practice guidelines (2012) published by the Japanese Society for Dialysis Therapy (JSDT) suggests GA levels < 20.0% as a potential target level for glycemic control in patients with diabetes on HD without a history of cardiovascular disease (CVD) and <24.0% for those with such a history^7^.

The issue of glycemic control may be particularly important in PD patients. However, few studies have examined the association between HbA1c and clinical outcomes in PD patients^8,9^, and, to our knowledge, no studies have investigated GA levels and mortality in PD patients. Accordingly, to establish the association between glycemic control and mortality—focusing on the ability to predict mortality with GA—we studied a nationwide registry of PD patients in Japan.

## Methods

### Patients

**Figure 1:**
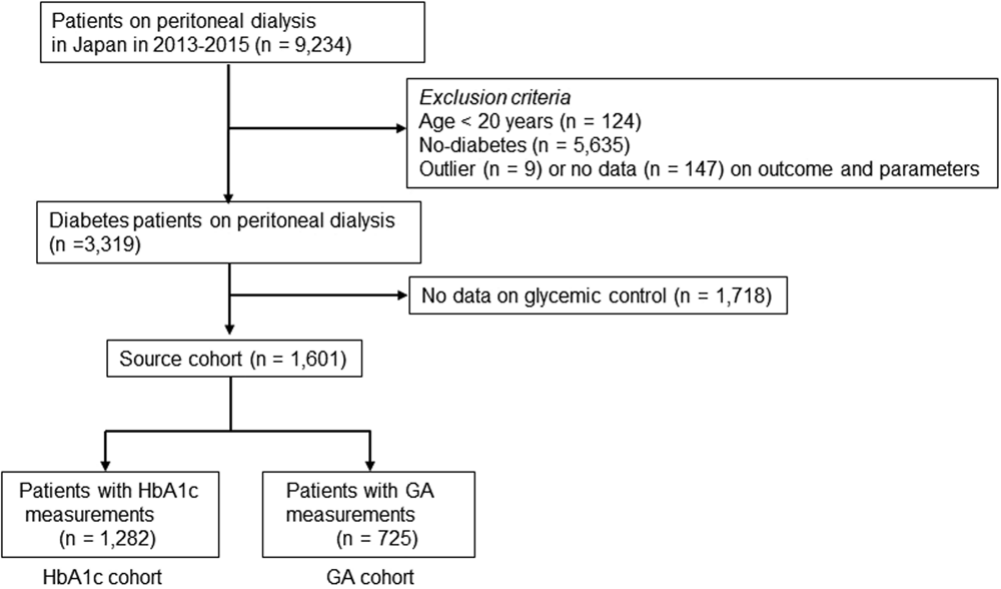
Flowchart of study participants.

The data were used in this study were from annual nationwide surveys of patients on dialysis conducted by JSDT and stored in the JSDT Renal Data Registry (JRDR). As described previously, all surveys were conducted by volunteers^10,11^. The standard analysis file prepared by the JRDR Committee for the present study included data for 314,438 patients who underwent dialysis at 4,268 facilities in the 2013 survey^12^, 320,448 patients dialyzed at 4,330 facilities in 2014, and 324,986 patients dialyzed at 4,321 centers in 2015^13^. Those diagnosed with diabetes and/or receiving diabetes medications and undergoing PD on 31 December 2013 were followed for 2 years. We excluded patients who had undergone HD, those aged <20 years, those whose records for date of birth, initiation of dialysis, or outcome were incomplete, and those who were considered outliers. In total, 315,631 patients on maintenance dialysis were registered at the end of 2013. After exclusions, 1,601 patients on PD were included in this study (Fig. 1). The HbA1c cohort comprised patients whose HbA1c levels were measured and included 1,282 patients after exclusions. The GA cohort comprised patients in whom glycated albumin (GA) levels were measured and included 725 patients after exclusions. HbA1c and GA levels were both measured in 413 patients, and these patients were included in both cohorts.

### Clinical and demographic measures

The following demographic and clinical data were collected: age, sex, duration of dialysis, primary cause of end-stage kidney disease (ESKD), body mass index (BMI), smoking status, use of antihypertensive agents, and history of complications of cardiovascular disease (CVD)—including cerebral infarction, cerebral hemorrhage, myocardial infarction, and limb amputation. Blood samples were collected and assayed at each dialysis facility or hospital, typically within 24 h of collection; and the latest values at the time of the survey were collated including serum albumin, hemoglobin, calcium, phosphate, intact parathyroid hormone (i-PTH), total cholesterol, high-density lipoprotein (HDL) cholesterol, and C-reactive protein (CRP). Using residual renal function (renal Kt/V) and peritoneal dialysis (PD Kt/V), weekly Kt/V urea values were recorded. The sum of renal Kt/V and PD Kt/V was considered as total Kt/V. The dialysate to plasma creatinine ratio (D/P Cr) was obtained using the peritoneal equilibration test. The type of PD fluid used (typically 2.5% glucose or icodextrin dialysate) and whether or not an automated PD system was used were also recorded. Furthermore, the type of treatment was recorded because PD + HD combination therapy, that is, HD treatment 1 day a week and PD on other days, has been approved in Japan since 2010.

We defined three categories of antidiabetic therapy: no medication, insulin therapy, and oral antidiabetic medication only. The no medication group comprised patients managed with diet modification therapy only or those for whom no antidiabetic medication was prescribed. Insulin therapy included insulin injection therapy only or a combination of insulin with an oral antidiabetic agent. Cause of death was classified as cardiovascular death, infection-related death, or other. We defined cardiovascular death as that due to chronic heart failure, pulmonary edema, ischemic heart disease (including acute myocardial infarction, i.e., death within 30 days of onset), arrhythmia, valvular heart disease, endocarditis, other cardiac disease, subarachnoid hemorrhage, intracerebral hemorrhage, cerebral infarction, or other brain disease.

### Statistical analysis

The data were expressed as proportions, using the mean ± standard deviation or median (interquartile range) as appropriate. We analyzed categorical variables using the chi-square test and continuous variables using the Student’s t-test. Categorical data were compared between groups with repeated-measures analysis of variance (ANOVA) and Tukey’s honestly significant difference test or Kruskal-Wallis test, as appropriate. Patients who were switched to HD during follow-up were censored from the outcome analysis. We defined reference ranges for the laboratory data such that patients with measured values outside of the following ranges were considered outliers and were excluded from the analysis: height 120–200 cm, body weight 20–150 kg, serum albumin 1.0–5.0 g/dL, CRP < 30 mg/dL, hemoglobin 5.0–20.0 g/dL, i-PTH < 3000 pg/mL, HbA1c 4.0–15.0%, and GA 10.0–50.0%. the conventional method for multivariate regression was used to imputed missing covariate data as appropriate.

We examined the clinical and demographic measures in each cohort by dividing the patients into 7 *a priori* categories defined by HbA1c values between <5.0% and ≥7.5% at increments of 0.5% (<5.0%, 5.0–5.4%, 5.5–5.9%, 6.0–6.4%, 6.5–6.9%, 7.0–7.4%, and ≥7.5%) and seven *a priori* categories defined by GA values of <12.0% and ≥22% at increments of 2% (<12.0%, 12.0–13.9%, 14.0–15.9%, 16.0–17.9%, 18.0–19.9%, 20.0–21.9%, and ≥22.0%). We used the 5.5–5.9% HbA1c category as the reference based on previous studies^14^. We used the 14.0–15.9% GA category as the reference because the standard value of GA was between 12.0 and 16.0%^15,16^. Survival analyses with Cox proportional hazards regression were used to examine whether basic factors at baseline—including age, sex, PD duration, cardiovascular comorbidity, PD-related factors, and nutritional and inflammatory factors—predicted survival for up to 2 years of follow-up. To examine the relationship between dialysis duration and risk of death, we grouped the patients into six *a priori* categories by duration of dialysis (<2, 2 to <4, 4 to <6, 6 to <8, 8 to <10, and ≥10 years). Additional analyses were performed with adjustment for PD-related factors, including residual renal function (i.e., anuric or non-anuric status), total Kt/V, use of 2.5% glucose or icodextrin dialysate, and type of treatment (i.e., user or non-user of an automated PD device, and PD alone or combination therapy with HD). Further analyses were performed with adjustments for nutrition-related and inflammation-related factors, including BMI and levels of hemoglobin, albumin, total cholesterol, HDL-cholesterol, calcium, phosphate, i-PTH, and CRP. Age, hemoglobin, and CRP levels were analyzed as continuous variables. We performed unadjusted and adjusted analyses that included all variables for all-cause mortality.

The study was conducted according to the principles of the Declaration of Helsinki, Japanese privacy protection laws, and Ethical Guidelines for Medical and Health Research Involving Human Subjects published by the Ministry of Education, Science and Culture, and the Ministry of Health, Labour and Welfare in 2015. The study protocol was approved by the Medicine Ethics Committee of the Japanese Society for Dialysis Therapy. The study was registered with the University Hospital Medical Information Network (UMIN000018641). The need for informed consent was waived because the study used de-identified information. All analysis was performed using JMP^®^ version 13.0 (SAS Institute, Cary, NC). P-values less than 0.05 were considered statistically significant.

## Results

### Patients demographic and clinical characteristics

Table 1 shows baseline demographic, clinical, and laboratory characteristics of diabetes patients with and without data on glycemic control (n = 1601 and 1718, respectively). Individuals with missing data were more likely to have diet therapy alone and combination therapy with HD, less likely to have antihypertensive agents, and have lower BMI. The differences in many of the other characteristics were not statistically significant (Table 1). The group of 1601 patients had the following characteristics: mean age 63.9 ± 11.6 years; 71.0% male; dialysis duration 27 (13–50) months; CVD history 24.7%; BMI 24.5 ± 3.9; serum albumin 3.2 ± 0.5g/dL; and hemoglobin 10.8 ± 1.3 g/dL. Baseline renal Kt/V, PD Kt/V, and total Kt/V were 0.4 (0–0.9), 1.3 ± 0.6, and 1.8 ± 0.8, respectively. Combination therapy with PD and HD was undertaken in 20% of the patients. An automated PD system was used in 42.9% of patients. During the 2-year study period, 173 patients (10.8%) died, 20 (1.2%) underwent kidney transplantation, and 499 (31.2%) were switched to HD; 909 (56.8%) patients were alive at the end of the study period.

**Table 1:** Demographic, clinical, and laboratory characteristics in patients with diabetes on PD with glycemic indices (n = 1601) and those without glycemic indices (n = 1718).

Variables	PD patients with HbA1c or GA measurement	PD patients without HbA1c or GA measurement	P value
n (male%)	1601 (71.0)	1718 (70.4)	0.483
Age, years	63.9 ± 11.6	64.0 ± 12.1	0.635
PD duration, m	27 [13–50]	25 [12–47]	0.163
Type 1 diabetes, %	8.9	7.7	0.172
CVD comorbidity, %	24.7	29.6	0.098
Smoking, %	11.2	9.5	0.214
Insulin, %	35.4	32.4	0.196
Oral antidiabetic agents, %	42.2	38.6	0.146
Diet therapy alone, %	34.5	42.2	0.001
Antihypertensive agents, %	83.5	79.9	0.023
HD combination, %	20.0	17.0	0.022
Anuric %	18.8	17.9	0.646
Total Kt/V	1.8 ± 0.8	1.8 ± 0.8	0.914
2.5% glucose dialysate use, %	41.6	37.0	0.043
Icodextrin user, %	47.2	42.6	0.052
D/P Cr	0.67 ± 0.13	0.66 ± 0.12	0.204
APD user, %	42.9	43.8	0.689
Body mass index, kg/m^2^	24.5 ± 3.9	24.1 ± 3.8	0.049
Hemoglobin, g/dL	10.8 ± 1.3	10.7 ± 1.4	0.251
Serum albumin, g/dL	3.2 ± 0.5	3.2 ± 0.6	0.349
Total cholesterol, mg/dL	172 ± 40	176 ± 40	0.062
HDL-cholesterol, mg/dL	46 ± 16	46 ± 17	0.752
C-reactive protein, mg/dL	0.16 [0.08–0.46]	0.20 [0.2–0.55}	0.106
Calcium, mg/dL	8.6 ± 0.8	8.6 ± 0.8	0.997
Phosphate, mg/dL	5.2 ± 1.3	5.2 ± 1.4	0.962
Intact-PTH, pg/mL	152 [76–249]	142 [80–247]	0.367

APD, automated peritoneal dialysis; CVD, cardiovascular disease; GA, glycated albumin; HD, hemodialysis; HDL, high-density lipoprotein; PD, peritoneal dialysis; PTH, parathyroid hormone.

### HbA1c cohort

Table 2 shows hazard ratios (HRs) in the unadjusted ANOVA evaluated as potential predictors of mortality in the HbA1c cohort. There was no significant effect of sex on patient survival (P = 0.185). Older age, longer duration of dialysis, and comorbid CVD were significant predictors of mortality. Lower dialysis dose, assessed by using total Kt/V, was found to be associated with higher risk of mortality. The use of 2.5% glucose dialysate and an anuric state at baseline were significant predictors of mortality. Poor nutritional status and a more severe inflammatory status, indicated by lower hemoglobin, higher CRP, lower serum albumin, and lower BMI, were also associated with higher mortality in patients on PD.

**Table 2:** Hazard ratios (with 95% CIs) for variables evaluated as potential predictors of all-cause mortality in the HbA1c cohort.

Variables	n (%)	HR	95% CI	P value
Male	919 (71.7)	1.00	Reference	Reference
Female	363 (28.3)	1.27	0.89–1.80	0.185
**Age**				
1 year increase	1282 (100)	1.05	1.03–1.06	<0.0001
**Duration of PD (year)**				
<2	588 (45.9)	1.00	Reference	Reference
2 ≤ < 4	365 (28.4)	1.93	1.28–2.91	0.002
4 ≤ < 6	196 (15.3)	2.89	1.81–4.61	<0.0001
6 ≤ < 8	84 (6.6)	2.64	1.32–5.31	0.006
8≤	49 (3.8)	2.19	0.94–5.08	0.067
**Type of diabetes**				
Type 2	1184 (92.4)	1.00	Reference	Reference
Type 1	98 (7.6)	0.78	0.40–1.52	0.472
**CVD comorbidity**				
No	967 (75.5)	1.00	Reference	Reference
Yes	313 (24.5)	2.65	1.86–3.77	<0.0001
**Smoking**				
No	1023 (89.0)	1.00	Reference	Reference
Yes	126 (11.0)	1.06	0.59–1.82	0.823
**Antihypertensive agent**				
User	994 (82.9)	1.00	Reference	Reference
Non-user	205 (17.1)	2.27	1.51–3.41	<0.0001
**HD combination**				
No	1028 (80.2)	1.00	Reference	Reference
Yes	254 (19.8)	0.89	0.58–1.39	0.615
**Residual renal function**				
Non-anuric	762 (81.8)	1.00	Reference	Reference
Anuric	169 (18.2)	1.64	1.05–2.50	0.030
**Type of dialysate**				
2.5% non-user	661 (59.7)	1.00	Reference	Reference
2.5% user	446 (40.3)	1.53	1.06–2.28	0.025
**Type of dialysate**				
Icodextrin non-user	615 (55.5)	1.00	Reference	Reference
Icodextrin user	492 (44.4)	1.01	0.69–1.45	0.988
**Type of treatment**				
APD non-user	641 (58.0)	1.00	Reference	Reference
APD user	464 (42.0)	0.71	0.49–1.01	0.053
**Total Kt/V**				
<1.1	45 (7.0)	2.73	1.02–7.29	0.043
1.1 ≤ < 1.4	78 (12.2)	1.62	0.65–4.06	0.297
1.4 ≤ < 1.7	128 (20.0)	1.00	Reference	Reference
1.7 ≤ < 2.0	170 (26.5)	0.91	0.41–2.05	0.825
2.0≤	220 (34.3)	0.71	0.32–1.56	0.395
**Body mass index (kg/m**^**2**^)				
<20.0	102 (9.3)	2.21	1.13–4.33	0.020
20 ≤ < 22	186 (16.9)	1.92	1.07–3.44	0.028
22 ≤ < 24	254 (23.1)	1.00	Reference	Reference
24 ≤ < 26	202 (18.4)	1.20	0.64–2.23	0.567
26 ≤ < 28	164 (14.9)	1.43	0.74–2.76	0.278
28≤	191 (17.4)	1.07	0.55–2.08	0.837
**Serum albumin (g/dL)**				
<2.5	87 (7.1)	5.41	2.99–9.78	<0.0001
2.5 ≤ < 3.0	258 (20.9)	2.25	1.45–3.47	0.0003
3.0 ≤ < 3.5	461 (37.4)	1.00	Reference	Reference
3.5 ≤ < 4.0	336 (27.3)	0.45	0.25–0.76	0.004
4.0≤	90 (7.3)	0.45	0.18–1.19	0.112
**Hemoglobin**				
1 g/dL increase	1253 (97.7)	0.81	0.71–0.94	0.005
**C-reactive protein**				
1 mg/dL increase	1126 (87.8)	1.46	1.26–1.70	<0.0001
**Total cholesterol (mg/dL)**				
<130	139 (13.4)	1.01	0.55–1.87	0.961
130 ≤ < 150	176 (17.0)	0.75	0.41–1.36	0.347
150 ≤ < 170	221 (21.3)	1.00	Reference	Reference
170 ≤ < 190	196 (18.9)	0.59	0.33–1.06	0.079
190 ≤ < 210	134 (12.9)	0.73	0.38–1.36	0.318
210≤	171 (16.5)	0.48	0.25–0.91	0.025
**HDL-cholesterol (mg/dL)**				
<30	123 (12.1)	1.44	0.80–2.59	0.219
30 ≤ < 40	262 (25.8)	0.87	0.52–1.45	0.601
40 ≤ < 50	267 (26.3)	1.00	Reference	Reference
50 ≤ < 60	169 (16.7)	0.62	0.33–1.17	0.145
60 ≤ < 70	94 (9.3)	0.67	0.31–1.43	0.307
70≤	99 (9.8)	0.48	0.21–1.08	0.079
**Calcium (mg/dL)**				
<8.0	261 (20.7)	1.47	0.92–2.35	0.104
8.0 ≤ < 8.5	271 (21.5)	1.13	0.69–1.85	0.606
8.5 ≤ < 9.0	332 (26.4)	1.00	Reference	Reference
9.0 ≤ < 9.5	227 (18.1)	0.65	0.37–1.15	0.142
9.5 ≤ < 10.0	118 (9.4)	0.76	0.35–1.67	0.502
10≤	49 (3.9)	1.92	0.88–4.19	0.100
**Phosphate (mg/dL)**				
<4.0	198 (15.5)	1.41	0.85–2.31	0.178
4.0 ≤ < 5.0	359 (28.1)	1.02	0.65–1.61	0.914
5.0 ≤ < 6.0	375 (29.4)	1.00	Reference	Reference
6.0 ≤ < 7.0	225 (17.6)	1.02	0.61–1.73	0.924
7.0 ≤ < 8.0	83 (6.5)	1.35	0.66–2.78	0.404
8.0≤	37 (2.9)	0.22	0.02–1.68	0.145
**Intact-PTH (pg/mL)**				
<60	199 (17.7)	1.40	0.82–2.38	0.215
60 ≤ < 140	306 (27.2)	0.87	0.51–1.46	0.601
140 ≤ < 220	269 (23.9)	1.00	Reference	Reference
220 ≤ < 300	147 (13.1)	1.25	0.68–2.31	0.457
300 ≤ < 380	83 (7.4)	0.64	0.26–1.52	0.311
380≤	120 (10.7)	0.86	0.43–1.74	0.688

CVD, cardiovascular disease; HD, hemodialysis; HDL, high-density lipoprotein; PD, peritoneal dialysis; PTH, parathyroid hormone.

Table 3 shows baseline data for demographics and clinical and laboratory characteristics of PD patients, based on the seven *a priori* categories according to baseline HbA1c. Higher HbA1c levels were noted to be associated with higher rates of type 1 diabetes and insulin use, 2.5% glucose dialysate, a lower proportion of males, and higher hemoglobin and CRP levels. The rate of transfer from PD to HD was not associated with the *a priori* HbA1c categories.

**Table 3:** Demographic, clinical, and laboratory values in 1282 PD patients, according to categories of HbA1c.

Variables	All	HbA1c Categories							P value
		<5.0	5.0 to 5.4	5.5 to 5.9	6.0 to 6.4	6.5 to 6.9	7.0 to 7.4	≥7.5	
n (male%)	1282 (71.7)	86 (65.1)	217 (80.1)	239 (79.5)	275 (71.3)	195 (64.6)	97 (65.0)	119 (59.7)	<0.0001
Age (years)	63.9 ± 11.6	61.8 ± 11.7	63.3 ± 11.4	63.5 ± 11.2	64.8 ± 11.7	65.2 ± 11.7	64.5 ± 11.7	63.2 ± 12.1	0.198
PD duration (m)	26 [12–49]	30 [16–57]	24 [10–49]	24 [12–49]	26 [11–49]	27 [13–46]	25 [12–48]	26 [14–47]	0.274
Type 1 (%)	7.6	5.8	5.9	5.9	6.6	8.2	13.4	13.5	0.037
CVD comorbidity (%)	24.4	23.3	20.7	22.7	27.3	24.6	23.7	30.2	0.437
Smoking (%)	10.9	8.9	12.7	9.2	12.7	13.5	3.2	14.4	0.259
Insulin (%)	35.4	11.8	21.1	25.2	35.3	50.0	52.8	62.8	<0.0001
Oral antidiabetic agents (%)	41.3	29.2	33.1	40.7	43.9	43.7	53.3	52.7	0.0006
Diet therapy alone (%)	35.5	62.3	51.6	41.9	32.1	19.7	13.9	17.5	<0.0001
Antihypertensive agents (%)	82.9	81.0	87.0	86.0	84.0	80.0	81.0	71.2	0.012
HD combination (%)	19.8	26.7	21.0	16.3	17.5	16.4	22.6	27.7	0.070
Anuric (%)	18.2	19.4	20.6	15.8	17.2	14.4	18.8	26.0	0.396
Total Kt/V	1.9 ± 0.9	2.1 ± 1.2	1.7 ± 0.7	1.9 ± 0.9	1.8 ± 0.6	2.0 ± 1.0	1.9 ± 0.6	2.0 ± 1.1	0.129
2.5% glucose dialysate use (%)	40.3	30.0	36.6	39.5	38.4	42.2	49.1	51.0	0.049
Icodextrin dialysate use (%)	55.6	44.3	45.1	40.0	47.9	42.8	45.5	47.9	0.722
D/P Cr	0.67 ± 0.13	0.68 ± 0.13	0.67 ± 0.15	0.69 ± 0.13	0.68 ± 0.12	0.66 ± 0.12	0.67 ± 0.13	0.62 ± 0.12	0.043
APD user (%)	41.9	46.4	42.7	42.8	41.0	42.2	45.2	34.7	0.776
Body mass index (kg/m^2^)	24.5 ± 3.9	24.7 ± 3.2	24.0 ± 3.5	24.2 ± 3.9	24.7 ± 3.8	25.1 ± 4.3	24.6 ± 4.3	25.2 ± 4.3	0.088
Hemoglobin (g/dL)	10.8 ± 1.3	10.2 ± 1.4	10.7 ± 1.3	10.7 ± 1.2	10.8 ± 1.3	10.9 ± 1.3	10.9 ± 1.4	10.9 ± 1.4	0.0009
Serum albumin (g/dL)	3.2 ± 0.5	3.2 ± 0.6	3.2 ± 0.5	3.3 ± 0.5	3.2 ± 0.6	3.2 ± 0.5	3.2 ± 0.6	3.2 ± 0.5	0.476
Total cholesterol (mg/dL)	172 ± 40	171 ± 43	165 ± 36	172 ± 42	170 ± 39	182 ± 41	172 ± 38	174 ± 41	0.015
HDL-cholesterol (mg/dL)	47 ± 16	50 ± 18	48 ± 17	49 ± 16	45 ± 15	44 ± 15	46 ± 17	42 ± 17	0.002
C-reactive protein (mg/dL)	0.15 [0.08–0.44]	0.14 [0.10–0.32]	0.12 [0.07–0.37]	0.11 [0.05–0.34]	0.19 [0.09–0.53]	0.16 [0.09–0.53]	0.22 [0.10–0.70]	0.21 [0.10–0.62]	0.017
Calcium (mg/dL)	8.6 ± 0.8	8.5 ± 0.7	8.5 ± 0.8	8.5 ± 0.7	8.5 ± 0.8	8.6 ± 0.8	8.7 ± 0.8	8.6 ± 0.8	0.409
Phosphate (mg/dL)	5.2 ± 1.3	5.3 ± 1.3	5.3 ± 1.3	5.2 ± 1.1	5.1 ± 1.2	5.1 ± 1.2	5.2 ± 1.3	5.4 ± 1.6	0.168
Intact-PTH (pg/mL)	153 [76–248]	158 [75–200]	151 [73–241]	138 [69–242]	153 [83–241]	151 [84–287]	172 [68–273]	193 [89–290]	0.246
Switch to HD, n (%)	396 (30.8)	24 (27.9)	68 (31.1)	97 (33.3)	85 (30.9)	58 (29.7)	31 (31.9)	33 (27.7)	0.886
Death (%)	18.4	12.1	18.2	16.6	18.7	18.7	21.7	23.6	0.635
Cardiovascular death	7.4	4.9	7.9	6.7	6.9	5.8	11.3	10.5	0.719
Infection-related death	5.1	0	5.2	3.5	5.3	6.6	5.2	9.3	0.275

CVD, cardiovascular disease; HD, hemodialysis; HDL, high-density lipoprotein; PD, peritoneal dialysis; PTH, parathyroid hormone.

Unadjusted all-cause death HRs and 95% CIs for HbA1c < 5.0%, 5.0–5.4%, 6.0–6.4%, 6.5–6.9%, 7.0–7.4%, and ≥ 7.5%, compared with 5.5–5.9% (reference), were 0.69 (0.31–1.53), 1.10 (0.67–1.81), 1.12 (0.68–1.84), 1.12 (0.65–1.92), 1.36 (0.72–2.55), and 1.51 (0.85–2.66), respectively.

**Figure 2:**
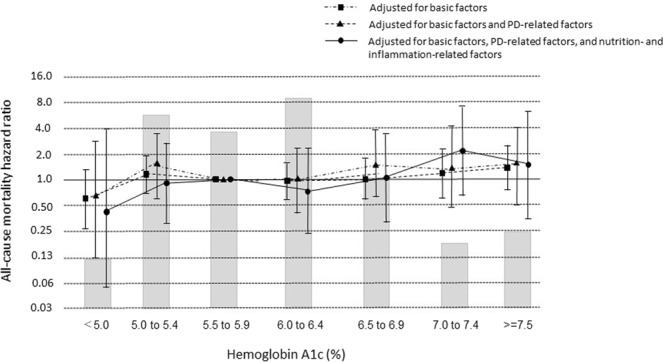
Hazard ratios for all-cause mortality over the entire range of hemoglobin A1c in 1282 patients undergoing peritoneal dialysis (PD), determined by Cox proportional hazards regression analysis. Basic factors include age, sex, dialysis duration, and presence or absence of cardiovascular comorbidity. PD-related factors include anuric or non-anuric state, total Kt/V, use of 2.5% glucose or icodextrin dialysate, and use of an automated PD system. Nutrition-related and inflammation-related factors include BMI and levels of hemoglobin, albumin, total cholesterol, HDL-cholesterol, calcium, phosphate, i-PTH, and CRP. Error bars correspond to 95% confidence intervals.

Figure 2 shows adjusted death HRs for groups based on HbA1c at baseline. All-cause death HR for each HbA1c category did not differ significantly compared with the reference HbA1c after adjusting separately for basic factors, after adjusting for basic factors plus PD-related factors, and after adjusting for basic factors plus PD- nutrition-, and inflammation-related factors.

### GA cohort

Table 4 shows hazard ratios (HRs) in the unadjusted ANOVA evaluated as potential predictors of mortality in the GA cohort. There was no significant effect of sex on patient survival. As in the HbA1c cohort, older age, longer duration of dialysis, comorbid CVD, lower total Kt/V, use of 2.5% glucose dialysate, and an anuric state at baseline were significant predictors of mortality. Lower hemoglobin, higher CRP, lower serum albumin, and lower BMI were also associated with higher mortality in patients on PD.

**Table 4:** Hazard ratios (with 95% CIs) for variables evaluated as potential predictors of all-cause mortality in the GA cohort.

Variables	n (%)	HR	95% CI	P value
Male	514 (70.9)	1.00	Reference	Reference
Female	211 (29.1)	1.13	0.71–1.78	0.608
**Age**				
1 year increase	725 (100)	1.05	1.02–1.07	<0.0001
**Duration of PD (year)**				
<2	310 (42.8)	1.00	Reference	Reference
2 ≤ < 4	204 (28.1)	1.80	1.06–3.06	0.029
4 ≤ < 6	116 (16.0)	2.03	1.02–4.03	0.043
6 ≤ < 8	57 (7.9)	3.17	1.36–7.37	0.007
8≤	38 (5.2)	2.27	0.88–5.85	0.087
**Type of diabetes**				
Type 2	637 (87.9)	1.00	Reference	Reference
Type 1	88 (12.1)	1.09	0.57–2.11	0.782
**CVD comorbidity**				
No	535 (73.8)	1.00	Reference	Reference
Yes	190 (26.2)	2.69	1.71–4.24	<0.0001
**Smoking**				
No	566 (89.0)	1.00	Reference	Reference
Yes	70 (11.0)	1.22	0.58–2.59	0.592
**Antihypertensive agent**				
User	551 (83.6)	1.00	Reference	Reference
Non-user	108 (16.4)	1.93	1.12–3.33	0.017
**HD combination**				
No	553 (76.3)	1.00	Reference	Reference
Yes	172 (23.7)	1.01	0.58–1.76	0.971
**Residual renal function**				
Non-anuric	429 (79.2)	1.00	Reference	Reference
Anuric	113 (20.8)	1.78	1.01–3.22	0.041
**Type of dialysate**				
2.5% non-user	367 (56.4)	1.00	Reference	Reference
2.5% user	284 (43.6)	2.19	1.34–3.59	0.002
**Type of dialysate**				
Icodextrin non-user	302 (46.4)	1.00	Reference	Reference
Icodextrin user	349 (53.6)	1.15	0.73–1.83	0.528
**Type of treatment**				
APD non-user	346 (53.7)	1.00	Reference	Reference
APD user	298 (46.3)	0.74	0.48–1.19	0.236
**Total Kt/V**				
<1.1	55 (11.9)	3.43	1.33–8.83	0.011
1.1 ≤ < 1.4	64 (13.8)	2.53	0.93–6.93	0.068
1.4 ≤ < 1.7	102 (22.0)	1.00	Reference	Reference
1.7 ≤ < 2.0	107 (23.1)	0.74	0.27–2.05	0.573
2.0≤	135 (29.2)	1.02	0.41–2.51	0.972
**Body mass index (kg/m**^**2**^)				
<20.0	62 (9.9)	2.68	1.25–5.77	0.011
20 ≤ < 22	102 (16.4)	1.15	0.53–2.94	0.708
22 ≤ < 24	153 (24.5)	1.00	Reference	Reference
24 ≤ < 26	123 (19.7)	0.76	0.35–1.64	0.488
26 ≤ < 28	84 (13.5)	0.94	0.41–2.19	0.895
28≤	100 (16.0)	0.67	0.28–1.58	0.364
**Serum albumin (g/dL)**				
<2.5	47 (6.6)	12.0	5.17–27.9	<0.0001
2.5 ≤ < 3.0	141 (19.9)	1.35	0.70–2.57	0.365
3.0 ≤ < 3.5	283 (40.0)	1.00	Reference	Reference
3.5 ≤ < 4.0	193 (27.3)	0.59	0.30–1.16	0.129
4.0≤	44 (6.2)	0.17	0.02–1.25	0.082
**Hemoglobin**				
1 g/dL increase	711 (98.1)	0.71	0.59–0.84	<0.0001
**C-reactive protein**				
1 mg/dL increase	646 (89.1)	1.39	1.18–1.64	<0.0001
**Total cholesterol (mg/dL)**				
<130	83 (13.5)	1.26	0.58–2.74	0.552
130 ≤ < 150	105 (17.2)	0.78	0.37–1.64	0.524
150 ≤ < 170	124 (20.3)	1.00	Reference	Reference
170 ≤ < 190	108 (17.7)	0.75	0.36–1.53	0.432
190 ≤ < 210	89 (14.5)	0.76	0.36–1.63	0.492
210≤	103 (16.8)	0.23	0.08–0.60	0.003
**HDL-cholesterol (mg/dL)**				
<30	70 (11.8)	3.18	1.49–6.79	0.003
30 ≤ < 40	176 (29.7)	0.84	0.41–1.69	0.633
40 ≤ < 50	148 (25.0)	1.00	Reference	Reference
50 ≤ < 60	89 (15.0)	1.12	0.49–2.51	0.791
60 ≤ < 70	59 (10.0)	0.44	0.14–1.39	0.165
70≤	50 (8.5)	0.41	0.11–1.50	0.179
**Calcium (mg/dL)**				
<8.0	154 (21.7)	0.72	0.29–1.75	0.473
8.0 ≤ < 8.5	159 (22.4)	0.96	0.45–2.03	0.918
8.5 ≤ < 9.0	185 (26.1)	1.00	Reference	Reference
9.0 ≤ < 9.5	116 (16.4)	0.76	0.34–1.68	0.508
9.5 ≤ < 10.0	66 (9.3)	0.78	0.29–2.09	0.629
10≤	29 (4.1)	1.83	0.64–5.18	0.253
**Phosphate (mg/dL)**				
<4.0	107 (14.8)	1.47	0.75–2.88	0.258
4.0 ≤ < 5.0	210 (27.8)	1.47	0.83–2.60	0.179
5.0 ≤ < 6.0	221 (30.5)	1.00	Reference	Reference
6.0 ≤ < 7.0	115 (15.9)	1.04	0.51–2.13	0.903
7.0 ≤ < 8.0	58 (8.0)	1.52	0.64–3.58	0.335
8.0≤	22 (3.0)	0.42	0.05–3.39	0.418
**Intact-PTH (pg/mL)**				
<60	119 (18.3)	1.42	0.61–3.34	0.412
60 ≤ < 140	181 (27.9)	1.23	0.53–2.84	0.617
140 ≤ < 220	151 (23.2)	1.00	Reference	Reference
220 ≤ < 300	88 (13.5)	1.17	0.48–2.85	0.718
300 ≤ < 380	44 (6.8)	0.63	0.18–2.12	0.461
380≤	67 (10.3)	1.71	0.52–5.57	0.368

CVD, cardiovascular disease; HD, hemodialysis; HDL, high-density lipoprotein; PD, peritoneal dialysis; PTH, parathyroid hormone.

Table 5 shows baseline data for demographics and clinical and laboratory characteristics of the PD patients based on the seven *a priori* categories according to baseline GA. Higher GA levels were noted to be associated with older age, type 1 diabetes, insulin use, 2.5% glucose dialysate, and higher CRP levels. The rate of transfer from PD to HD was not associated with *a priori* GA categories. A higher GA level was associated with higher mortality, especially infection-related death.

**Table 5:** Demographic, clinical, and laboratory values in 725 PD patients, according to categories of GA.

Variables	All	GA Categories							P value
		<12.0	12.0 to 13.9	14.0 to 15.9	16.0 to 17.9	18.0 to 19.9	20.0 to 21.9	≥22	
n (male%)	725 (70.1)	42 (61.9)	132 (71.2)	164 (79.3)	150 (68.0)	100 (71.0)	63 (66.7)	74 (66.2)	0.153
Age (years)	63.5 ± 11.7	58.5 ± 12.2	61.7 ± 11.9	64.4 ± 11.9	64.4 ± 11.4	63.6 ± 11.6	63.8 ± 11.0	65.0 ± 10.9	0.028
PD duration (m)	29 [14–53]	27 [15–54]	24 [13–49]	24 [13–49]	33 [14–55]	37 [19–64]	29 [12–57]	27 [13–49]	0.166
Type 1 (%)	12.1	4.7	5.3	9.7	14.0	19.0	14.3	18.9	0.006
CVD comorbidity (%)	25.8	16.7	22.7	27.4	26.7	30.0	17.5	32.4	0.241
Smoking (%)	11.0	13.5	10.9	6.9	11.5	17.2	5.5	14.0	0.193
Insulin (%)	37.5	30.5	20.6	27.6	39.2	47.4	50.0	65.1	<0.0001
Oral antidiabetic agents (%)	43.3	37.1	36.3	46.5	41.2	49.5	45.3	46.2	0.478
Diet therapy alone (%)	32.0	38.9	47.5	37.2	31.8	20.8	19.6	15.9	<0.0001
Antihypertensive agents (%)	83.6	84.2	87.0	84.6	84.8	76.9	89.1	76.9	0.293
HD combination (%)	23.7	21.4	19.7	23.1	24.6	34.0	15.8	24.3	0.163
Anuric (%)	20.8	13.9	15.7	18.0	25.0	19.4	21.7	33.9	0.131
Total Kt/V	1.7 ± 0.7	1.7 ± 0.6	1.7 ± 0.6	1.7 ± 0.6	1.8 ± 0.9	1.6 ± 0.7	1.9 ± 0.9	1.6 ± 0.6	0.224
2.5% glucose dialysate use (%)	43.6	32.4	35.8	34.9	45.9	61.3	45.6	52.9	0.0007
Icodextrin dialysate use (%)	53.6	54.1	60.0	54.1	51.1	46.6	52.6	55.9	0.643
D/P Cr	0.67 ± 0.13	0.71 ± 0.14	0.69 ± 0.13	0.66 ± 0.15	0.66 ± 0.12	0.67 ± 0.11	0.66 ± 0.13	0.67 ± 0.11	0.273
APD user (%)	46.2	44.7	43.3	43.4	47.7	57.3	42.1	44.8	0.439
Body mass index (kg/m^2^)	24.3 ± 3.7	25.1 ± 3.7	24.9 ± 3.9	24.1 ± 3.4	24.3 ± 3.6	23.9 ± 3.7	24.7 ± 4.6	23.4 ± 3.5	0.108
Hemoglobin (g/dL)	10.8 ± 1.4	10.8 ± 1.3	10.7 ± 1.3	10.9 ± 1.4	10.8 ± 1.4	10.6 ± 1.4	11.0 ± 1.4	10.6 ± 1.4	0.572
Serum albumin (g/dL)	3.2 ± 0.5	3.2 ± 0.5	3.2 ± 0.5	3.3 ± 0.5	3.2 ± 0.5	3.2 ± 0.6	3.3 ± 0.4	3.2 ± 0.6	0.391
Total cholesterol (mg/dL)	172 ± 40	170 ± 43	173 ± 34	173 ± 41	172 ± 41	168 ± 41	174 ± 43	170 ± 41	0.962
HDL-cholesterol (mg/dL)	46 ± 17	50 ± 23	47 ± 14	45 ± 14	47 ± 20	43 ± 14	45 ± 16	46 ± 24	0.351
C-reactive protein (mg/dL)	0.17 [0.07–0.50]	0.12 [0.08–0.42]	0.17 [0.08–0.38]	0.15 [0.07–0.44]	0.17 [0.09–0.48]	0.17 [0.07–0.62]	0.11 [0.05–0.46]	0.35 [0.09–1.01]	0.003
Calcium (mg/dL)	8.5 ± 0.8	8.5 ± 0.8	8.6 ± 0.9	8.6 ± 0.8	8.6 ± 0.8	8.5 ± 0.8	8.6 ± 0.8	8.4 ± 0.9	0.498
Phosphate (mg/dL)	5.3 ± 1.3	5.6 ± 1.6	5.4 ± 1.3	5.3 ± 1.4	5.3 ± 1.2	5.1 ± 1.2	5.0 ± 1.3	5.2 ± 1.3	0.285
Intact-PTH (pg/mL)	152 [74–246]	151 [79–207]	148 [68–236]	155 [75–257]	128 [67–232]	138 [68–235]	194 [85–249]	198 [81–295]	0.101
Switch to HD, n (%)	227 (31.3)	11 (26.2)	46 (34.8)	40 (24.4)	53 (35.3)	37 (37.0)	22 (34.9)	18 (24.3)	0.215
Death (%)	15.9	12.9	12.8	12.9	16.5	14.3	14.6	30.3	0.002
Cardiovascular death	7.1	10.0	7.4	6.1	5.8	5.3	7.9	11.4	0.886
Infection-related death	7.1	0	3.9	5.3	9.0	6.9	5.4	18.8	0.025

CVD, cardiovascular disease; HD, hemodialysis; HDL, high-density lipoprotein; PD, peritoneal dialysis; PTH, parathyroid hormone.

**Figure 3:**
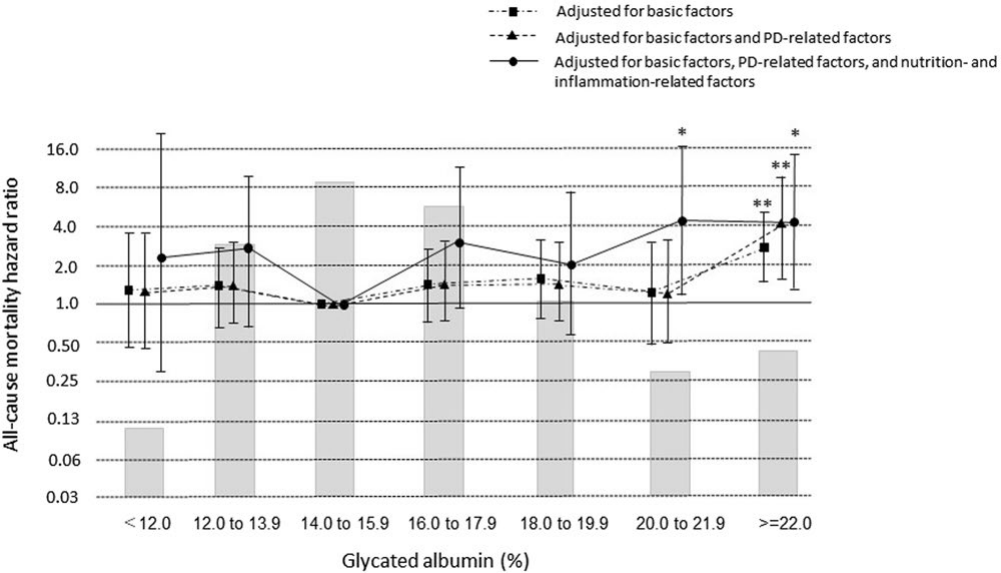
Hazard ratios for all-cause mortality over the entire range of glycated albumin in 725 patients undergoing PD, determined by Cox proportional hazards regression analysis. Basic factors include age, sex, dialysis duration, and presence or absence of cardiovascular comorbidity. PD-related factors include anuric or non-anuric state, total Kt/V, use of 2.5% glucose or icodextrin dialysate, and use of an automated PD system. Nutrition-related and inflammation-related factors include BMI and levels of hemoglobin, albumin, total cholesterol, HDL-cholesterol, calcium, phosphate, i-PTH, and CRP. *P < 0.05, **P < 0.01 vs. GA 14.0 to 15.9% (reference). Error bars correspond to 95% confidence intervals.

Unadjusted all-cause death HRs and 95% CIs for GAs < 12.0%, 12.0–13.9%, 16.0–17.9%, 18.0–19.9%, 20.0–21.9%, and ≥22.0%, compared with the reference value, were 1.14 (0.42–3.09), 1.17 (0.58–2.38), 1.50 (0.79–2.86), 1.68 (0.84–3.37), 1.22 (0.51–2.94), and 3.09 (1.66–5.77), respectively. Patients with GA ≥ 22.0% had a significantly higher death HR when compared with the reference GA (P = 0.0003). Figure 3 shows adjusted death HRs for groups based on GA at baseline. The all-cause death HR for GA ≥ 22.0%, compared with the reference GA, was 2.69 (1.43–5.03) after adjusting for basic factors and 4.09 (1.56–10.7) after adjusting for basic factors plus PD-related factors (P = 0.0018 and 0.0025, respectively). However, the all-cause death HR for GA categories 20.0–21.9% and ≥22.0% were 4.36 (1.10–17.0) and 4.10 (1.2–14.0) compared with the reference value after adjusting for basic factors plus PD-, nutrition-, and inflammation-related factors (P = 0.040 and 0.017, respectively).

## Discussion

In this study, we first identified the predictors of 2-year mortality in PD patients. Because survival outcome among dialysis patients might be determined by additional multiple confounding factors—dialysis-related or non-dialysis related—investigations into the control of these factors are difficult to perform. However, we compared mortality rates among glycemic control categories, adjusting for multiple potentially confounding factors. After fully adjusting for these factors, there were no significant differences for death HRs among the HbA1c categories compared with the HbA1c reference value of 5.5–5.9%. However, in this large-scale contemporary cohort of 1,601 PD patients, GA ≥ 20.0% was associated with higher all-cause mortality. This is the first study to suggest that mortality risk for PD patients might differ according to GA level.

Data from the US Renal Data System for 2010 revealed that the overall 1- and 2-year survival rates of patients on PD were 83% and 67.2%, respectively, whereas those of a diabetes subgroup were 80.3% and 61.7%^17^. A further study from Canada reported 1- and 2-year survival rates of 94% and 89% for patients without diabetes, respectively, and 91% and 76% for patients with diabetes^18^. Data from single centers in Asia and Europe also showed that survival rates were significantly lower in patients with diabetes on PD than in those without diabetes on PD^19^. In the present study, the 1- and 2-year survival rates were 88.3% and 84.0%, respectively, which are higher than those reported previously. Older age, longer duration of PD, use of 2.5% glucose dialysate, anuric state, and lower albumin levels and BMI at baseline might have contributed to lower survival rates in this cohort.

Relatively few studies have investigated PD patients with diabetes^8,9,20–22^. In the largest of the previous studies, which involved 2,798 patients, time-averaged HbA1c ≥ 8% was found to be associated with the highest risk of all-cause mortality^9^. Another study found that glycemic control was more important in patients who were less than 60 years old^20^. Infection was a notable cause of death among these patients, particularly those with HbA1c ≥ 8%^23^. These findings suggest that in PD patients, poor glycemic control adversely impacts survival due to an increased risk of infection. Similar results were obtained in a prospective observational study of diabetes patients on PD; non-cardiovascular mortality primarily due to infection was highest in patients in the highest HbA1c tertile^24^. The present study revealed no significant differences in the rates of cardiovascular death and infection-related death among the HbA1c categories. However, infection-related death occurred most frequently in patients in the highest GA category.

A meta-analysis reported that high levels of HbA1c (>8.5%) and very low HbA1c levels (<5.4%) were associated with an increased mortality risk^25^. The Dialysis Outcomes and Practice Patterns Study demonstrated that the relationship between low HbA1c and mortality appeared to be even stronger in patients with indicators of poor nutritional status, including low serum albumin, low BMI, or presence of cachexia^26^. These data suggest that not only high HbA1c levels but also low HbA1c levels related to malnutrition or anemia are associated with increased mortality in HD patients. Potential contributors to these phenomena include reduced kidney gluconeogenesis, decreased kidney and hepatic insulin clearance, diminished food intake, deficient catecholamine release, protein-energy wasting, and the hypoglycemic effects of HD. Furthermore, patients on dialysis have low HbA1c levels due to renal anemia and use of ESAs. Thus, HbA1c levels could overestimate glycemic control in HD patients and PD patients as well, because of renal anemia and ESA use in PD patients. In contrast, GA is not affected by hemoglobin levels and ESA^2–4^. Understanding factors associated with mortality remains a priority in clinical care for patients with diabetes on dialysis. Glycemic control is definitely the most important factor for diabetes patients, including those with ESKD. Recent reports have questioned the utility of HbA1c and GA as glycemic markers in HD patients^27,28^. Although GA may be a more accurate index of glycemic control than HbA1c in HD patients, there is limited evidence of an association between GA and mortality in PD patients. Therefore, concluding that GA is superior to HbA1c as a mortality marker in these patients is premature, although some data suggest that GA is less affected by anemia and serum albumin levels than HbA1c is. Nevertheless, the present study does provide some evidence for the utility of GA in predicting 2-year mortality in a large cohort of PD patients.

According to numerous studies, higher GA levels are associated with higher all-cause or cardiovascular mortality rates in HD patients with diabetes^29–31^. Remarkably, no significant association was seen between mean HbA1c levels and mortality in these patients. In addition, it was reported that GA, not HbA1c, accurately predicted the risk of death and hospitalization in patients with diabetes on HD (n = 401) or PD (n = 43)^32^. GA level is a useful index of glycemic control in HD patients, there is little evidence on the relationship between GA levels and the risk of cardiovascular events or prognosis in PD patients.

This study has several limitations. First, as in any annual survey or observational cohort study, sample size and power were limited in the present study. The number of patients who had higher HbA1c or GA categories was small and mortality was low compared with previous reports^13,27^; an adequate sample size was estimated to be 856 and 1905 patients in the GA cohort and HbA1c cohort, respectively. It must be noted that GA is not a widely available index, and there are limited outcome studies that used GA. Further studies are thus needed to confirm reproducibility. Second, as with any annual survey, our database involves a yearly one-point estimate of glycemic control, rendering analyses with time-averaged GA levels within a year impossible. However, some previous large-database reports have shown similar mortality among initial and time-averaged HbA1c groups^26,33^. Third, we lacked information about the use of ESA. However, because GA is reported to be unaffected by the life-span of erythrocytes or ESA administration^4,34,35^, the effect of ESA in the GA-mortality association may be small and hence may not change our results. Finally, blood glucose level data were not available and many patients in this study had either HbA1c or GA measured, not both. Therefore, we were unable to compare GA, HbA1c, and plasma glucose levels. Thus, in the future, prospective studies are warranted to elucidate target GA levels, given that several recommendations for the treatment of diabetes in PD patients are based on longer-term studies of HbA1c levels.

In conclusion, the findings of this study show that a GA > 20.0% was associated with decreased survival, especially infection-related death, in patients with diabetes on PD. No associations were evident between the HbA1c levels and 2-year mortality in PD patients. These results underscore the need for further research on the factors that influence patient outcomes, so as to identify alternative interventions that would improve the outlook for patients undergoing PD.
